# Ring-chain synergy in ionic liquid electrolytes for lithium batteries†
†Electronic supplementary information (ESI) available: Experimental methods, ATR-FTIR spectra and ionic conductivity with error bar of all the electrolyte systems. See DOI: 10.1039/c5sc02761f


**DOI:** 10.1039/c5sc02761f

**Published:** 2015-09-18

**Authors:** Feng Wu, Qizhen Zhu, Renjie Chen, Nan Chen, Yan Chen, Li Li

**Affiliations:** a Beijing Key Laboratory of Environmental Science and Engineering , School of Materials Science and Engineering , Beijing Institute of Technology , Beijing , 100081 , China . Email: chenrj@bit.edu.cn ; Fax: +86-10-68451429; b Collaborative Innovation Center of Electric Vehicles in Beijing , Beijing , 100081 , China

## Abstract

Lithium-ion batteries have been attracting much attention which enables the revolution of wireless global communication. Ionic liquids are regarded as promising candidates for lithium-ion battery electrolytes because they can overcome the limitations of high operating temperatures and flammability concerns of traditional electrolytes. However, at low temperatures they suffer from low ionic conductivity and phase transition. In this paper mixed electrolyte systems are described based on *N*-methoxyethyl-*N*-methylpyrrolidinium bis(trifluoromethanesulfonyl)-imide (Pyr_1,2O1_TFSI) and lithium difluoro(oxalate)borate (LiODFB) lithium salt, with ethylene sulphite (ES) or dimethyl sulphite (DMS) as a cosolvent. The mixed electrolyte system exhibits good ion transport properties (a conductivity of 8.163 mS cm^–1^), a wide electrochemical window (5.2 V), non-flammability, the ability to form films to protect the anode and a large operating temperature range (–40 °C to 60 °C). We compare the performance and function of the new mixed electrolyte system with a variety of ionic liquid/cosolvent electrolyte systems developed in previous studies. The ring-chain synergy takes advantage of the availability of both high permittivities based on the ring-like components and low viscosities based on the chain-like components in the mixed electrolyte system and causes the electrolyte to exhibit a good overall performance in safety, ion transport and compatibility with electrodes.

## Abstract 2

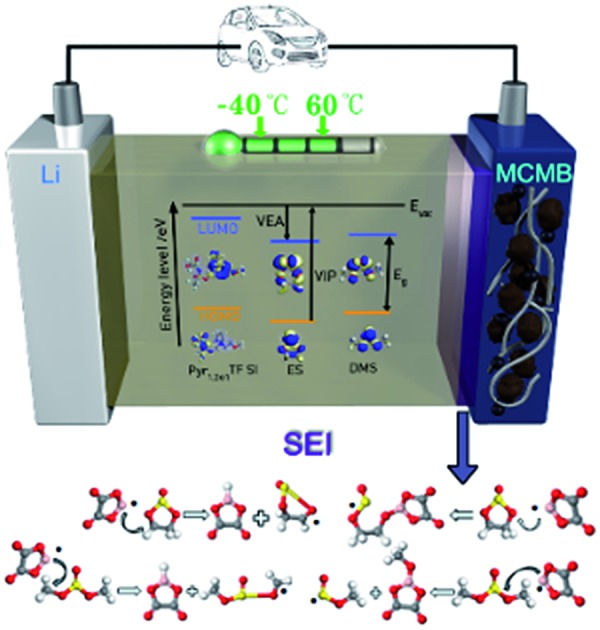
The ionic liquid-based electrolyte containing ring-like Pyr_1,2O1_TFSI and chain-like DMS exhibits good overall performance in lithium-ion batteries.

## Introduction

With increasing global concerns about climate change and the environment and a growing scarcity of traditional fossil fuels, substantial efforts have been focused on the development of new and renewable energy sources.^1^,^2^ Lithium-ion batteries are important as storage and conversion devices, which have a high energy density, long lifecycle, and significant environmental benefits.^3^,^4^ However, the adoption of lithium-ion battery technology still faces major challenges because the batteries can only operate within a narrow temperature range and because they present safety issues, namely inflammability.^5^–^7^ As a key component of a battery, the electrolyte has a potent influence on both of these issues, so the solution lies in improving/tailoring the electrolyte.^8^,^9^

In lithium-ion batteries, a variety of liquid solvents can be used as electrolytes to produce batteries with excellent ionic conductivity, and the use of organic carbonates as solvents for electrochemical applications is well established.^10^ However, these carbonates are highly flammable, posing a safety problem for large-scale applications. Ionic liquids, which have been the focus of significant research,^11^–^19^ are regarded as one of the most promising alternatives to the carbonates used as electrolytes in lithium batteries because of their high electrochemical and thermal stability. One challenge with ionic liquids, however, is that they tend to have a high viscosity, which can lead to decreased ionic conductivity in batteries. To overcome this problem, ionic liquid/organic solvent binary electrolyte systems, which combine the nonflammability of the ionic liquid and the low viscosity of the organic solvent, have been prepared.^14^,^15^,^20^,^21^ The addition of a small amount of carbonate in alkyl ammonium significantly improves the conductivity, reaching 12 mS cm^–1^ at 40 °C.^22^ By introducing 20% diethyl carbonate as a co-solvent into a pure ionic liquid electrolyte, the discharge capacity of the LiCoO_2_/Li cells reaches up to 102 mA h g^–1^ at 10 °C with negligible damage to the safety characteristics.^23^

**Fig. 1:**
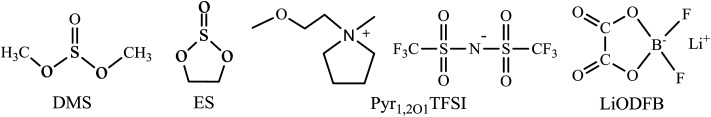
Molecular structures of the mixture components: dimethyl sulphite (DMS), ethylene sulphite (ES), *N*-methoxyethyl-*N*-methylpyrrolidinium bis(trifluoromethanesulfonyl)-imide (Pyr_1,2O1_TFSI) and lithium difluoro(oxalate)borate (LiODFB).

In this study, we report the preparation and electro-chemical evaluation of a mixed electrolyte system that takes advantage of the ring-like ionic liquid *N*-methoxyethyl-*N*-methylpyrrolidinium bis(trifluoromethanesulfonyl)-imide (Pyr_1,2O1_TFSI). The ether-functionalised cation (Fig. 1) effectively decreases the viscosity and provides an amorphous system below room temperature.^24^–^27^ Pyr_1,2O1_TFSI and EC/DEC binary mixtures with a volume ratio of 60 : 40 with LiTFSI demonstrated remarkably reduced flammability, ionic conductivity exceeding 7 mS cm^–1^ at 20 °C and an overall electrochemical window of 4.5 V.^15^ Sulphites are chosen as the cosolvents for their low viscosity, low melting point and outstanding ability to form effective solid electrolyte interface (SEI) layers to protect the electrodes.^28^–^32^ LiODFB is used as the lithium salt because of its good passivation toward Al^33^,^34^ to avoid corrosion of the Al current collector from the TFSI^–^ anion. The Pyr_1,2O1_TFSI/sulphite binary electrolytes were combined with LiODFB as the lithium salt.

So far, there has been no suitable single solvent available that simultaneously possesses a high dielectric permittivity, low viscosity and good compatibility with both an anode and cathode. As a compromise, the majority of the most common electrolyte systems used in lithium-ion batteries is a mixture of the ring-like ethylene carbonate with high dielectric permittivity or the chain-like carbonate with low viscosity. The combination of the beneficial effects of the carbonate species with atoms arranged in both rings and chains can improve the performance of the bulk electrolyte systems, which is designated as ring-chain synergy. However, key questions about the electrolyte performance remain unanswered. Can ring-chain synergy also be applicable for ionic liquid electrolytes? What is the optimal composition of an ionic liquid/organic cosolvent-based electrolyte that meets the demands in terms of safety, ion transport, and compatibility with the both cathodes and anodes in a lithium-ion battery? In order to answer these questions, this paper compares the improvement offered by ring-like ethylene sulphite (ES) and chain-like dimethyl sulphite (DMS) in electrochemical and physicochemical performance for the electrolyte based on Pyr_1,2O1_TFSI. Furthermore, we summarise and discuss the properties and performance of ionic liquid/cosolvent electrolyte systems derived both in our new research outlined here and in previous studies.

## Results, discussion and experimental

### Electrolyte properties

Solvation of Li^+^ in the electrolyte depends upon the interaction of Li^+^ and the solvent molecules.^35^ The Fourier transform infrared spectroscopy (FTIR) spectra of the electrolyte systems in which LiODFB and sulphites are added into Pyr_1,2O1_TFSI (Fig. S1†) are shown to investigate the Li^+^ solvation which depends on the intensity of interaction between LiODFB and the solvent molecules. The electron cloud density of –SO_3_ in DMS is more negative than that in ES. Affected by the coordination between Li^+^ and the electronegative O atom, the stretching vibrations of S=O become weaker. Consequently, the bond energy of O–S–O becomes stronger and the peak is red shifted to a lower frequency. The O–S–O stretching bands varying in ES (910 cm^–1^ and 654 cm^–1^) and DMS (953 cm^–1^ and 692 cm^–1^) indicates that the enhancement of Li^+^ solvation is more obvious in DMS.

**Fig. 2:**
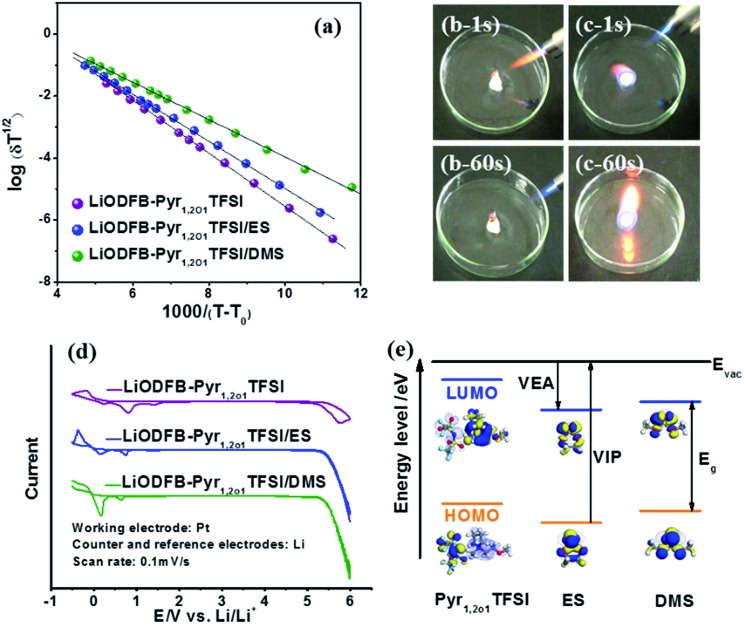
(a) VTF plots of the conductivity of LiODFB–Pyr_1,2O1_TFSI electrolytes with and without sulphites at various temperatures. Burning tests of (b) LiODFB–Pyr_1,2O1_TFSI/DMS and (c) LiPF_6_–EC/DEC. (d) CVs of LiODFB–Pyr_1,2O1_TFSI electrolytes with and without sulphite. (e) The energy level diagram of frontier molecular orbitals relative to the energy in a vacuum (*E*_vac_) and HOMO and LUMO plots of Pyr_1,2O1_TFSI, ES and DMS.

The ionic conductivity and the Arrhenius plots of the ionic conductivity of the LiODFB–Pyr_1,2O1_TFSI electrolyte with and without sulphites over a temperature range of –40 °C to 80 °C are given in Fig. 2(a) and Table 1. Although the ether group, as a lateral substituent, decreases the viscosity of the ionic liquid, the conductivity of the ionic liquid (1.9 mS cm^–1^) is not yet comparable to that of traditional carbonate electrolytes (5–10 mS cm^–1^).^33^ However, when Pyr_1,2O1_TFSI is mixed with sulphites, a significant increase (3–4 times) in the ionic conductivity is observed. LiODFB–Pyr_1,2O1_TFSI/DMS possesses an ionic conductivity at room temperature of 8.163 mS cm^–1^ with a relative standard departure of 4.14% among permitted error bars after testing 5 times (Fig. S2†). The conductivity value is close to that of carbonate-based ionic liquid electrolytes (8 mS cm^–1^).^15^ Fig. S3† shows the differential scanning calorimetry (DSC) curves of the electrolytes. LiODFB–Pyr_1,2O1_TFSI exhibits a large exothermal crystallisation peak (at –43 °C) due to incomplete crystallization of the sample prior to the DSC heating scan. It becomes lower (–51 °C) when DMS is introduced, which suggests that the addition of DMS makes the electrolyte useful at low temperatures.

**Table 1:** Ionic conductivity and VTF parameters relating to the conductivity of the electrolyte

Electrolyte system		LiODFB–Pyr_1,2O1_TFSI	LiODFB–Pyr_1,2O1_TFSI/ES	LiODFB–Pyr_1,2O1_TFSI/DMS
Ionic conductivity/mS cm^–1^	–40 °C	5.26 × 10^–3^	2.06 × 10^–1^	4.63 × 10^–1^
	–20 °C	8.40 × 10^–2^	9.63 × 10^–1^	1.50
	0 °C	4.91 × 10^–1^	2.69	3.80
	25 °C	1.91	6.06	8.16
	60 °C	6.66	14.0	16.6
VTF parameters	*A*/S cm^–1^ K^–1/2^	17.1	12.7	8.1
	*B*/K	835	748	609
	*T*_0_/°C	–109	–131	–125

The relationship between temperature and ionic conductivity is described by the Vogel–Tamman–Fulcher (VTF) equation,^36^,^37^

1

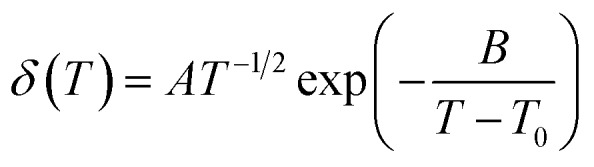

where *A* is assumed to be proportional to the number of carrier ions and *T*_0_ is the temperature at which the configurational entropy becomes zero. The VTF parameters for this system are presented in Table 1. *B* relating to the apparent activation energy shows the energy to overcome association among the ions of the ionic liquid to become free conductive ions. The modest ion association in the ionic liquid owing to the resultant effect of electrostatic forces, hydrogen bonds and van der Waals force brings about ion pairs and ion networks causing high viscosity and low conductivity. The large van der Waals force due to the long side-chain in Pyr_1,2O1_^+^ and strong hydrogen bond resulting from TFSI^–^ with a big size leads to the high apparent activation energy. The addition of sulphites with good dielectric properties and a low viscosity contributes to the dissociation between the positive and negative ions in the ionic liquid, resulting in *B* with relatively low value. The *T*_0_ of LiODFB–Pyr_1,2O1_TFSI/DMS is –125 °C, which is lower than that of ionic liquid electrolytes with carbonates (–103 °C),^15^ leading to a lower glass transition temperature and better low temperature performance.

To clarify the influence of the sulphites on electrochemical stability, the cyclic voltammograms (CVs) determining the oxidation/reduction potentials of the electrolytes are shown in Fig. 2(d). When the potential is increased to 5.2 V, the decomposition of LiODFB–Pyr_1,2O1_TFSI becomes significant, suggesting oxidative decomposition at a potential of 5.2 V (as compared to Li/Li^+^). The addition of sulphites only has a small influence on the oxidation potentials of the electrolytes; for all cases the oxidation potential exceeded 5 V (*vs.* Li/Li^+^). On the basis of molecular orbital theory, the difference between the calculated energies of the lowest unoccupied molecular orbital (LUMO) and the highest occupied molecular orbital (HOMO) determine the vertical electron affinity (VEA) and the vertical ionisation potential (VIP).^38^,^39^ As shown in Fig. 2(e), the LUMO and HOMO of ES have lower energies than those of DMS, indicating that ES has higher oxidation and reduction potentials.

The flammability of the electrolytes based on either ionic liquids or carbonates was tested, and the results are shown in Fig. 2(b) and (c) and S4.† The traditional carbonate electrolyte ignited immediately, while the ionic liquid electrolytes, including those with sulphites, did not ignite when exposed to a flame of 1300 °C for 60 s. This excellent flame resistance makes ionic liquid electrolytes a promising safe choice for use in lithium-ion batteries.

### Electrochemical compatibility with electrodes

**Fig. 3:**
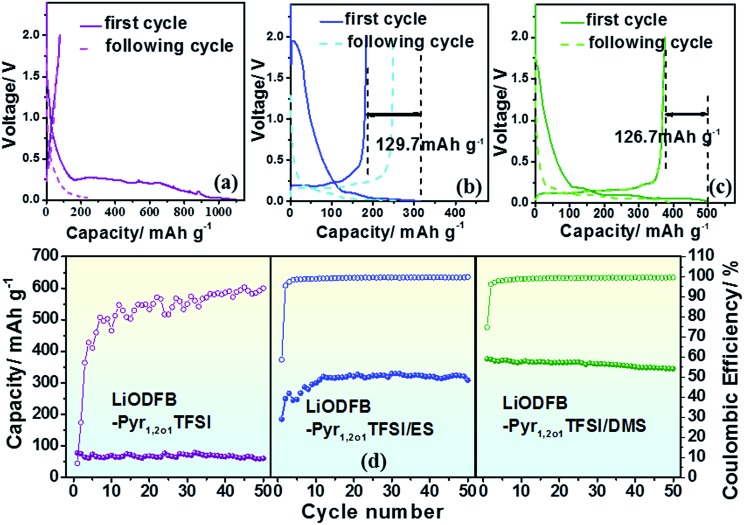
First and subsequent charge and discharge curves of Li/MCMB cells with (a) LiODFB–Pyr_1,2O1_TFSI, (b) LiODFB–Pyr_1,2O1_TFSI/ES or (c) LiODFB–Pyr_1,2O1_TFSI/DMS electrolytes. (d) Cycling performance and coulombic efficiency of the cells with the three electrolyte samples.

A mesocarbon microbead (MCMB) is chosen as the electrode to evaluate the electrochemical properties of cells with the electrolytes because it is abundant, inexpensive and has a high specific energy. In Fig. 3(a)–(c), the galvanostatic charge–discharge profiles of Li/MCMB cells with LiODFB–Pyr_1,2O1_TFSI do not feature a stable plateau, and the CV (Fig. S5†) without symmetrical oxidation/reduction peaks implies irreversible Li^+^ insertion/extraction. Li/MCMB cells containing electrolytes with sulphites show two discharge voltage plateaus in the initial cycle. The small plateau at 1.8 V in the first cycle disappearing in the following cycle may be mainly assigned to the formation of a SEI, leading to loss of capacity, and the smooth plateau at 0.005–0.4 V indicates a good reversibility in the Li^+^ insertion/extraction performance. The CVs in Fig. S5(b) and (c)† show reduction peaks at about 1.7 V indicating SEI forming in the first cycle and symmetrical oxidation/reduction peaks below 0.5 V which are in good agreement with the galvanostatic charge–discharge results.

The cycling performances of the Li/MCMB cells containing the electrolytes are demonstrated in Fig. 3(d). The cells containing LiODFB–Pyr_1,2O1_TFSI exhibit a limited reversible charge capacity below 100 mA h g^–1^. The initial discharge of cells with LiODFB–Pyr_1,2O1_TFSI/ES are about 313.9 mA h g^–1^. Compared with ES, the cells upon addition of DMS acquire a higher charge capacity of 376.2 mA h g^–1^ in the first cycle and retain a value of 345.0 mA h g^–1^ after 50 cycles. The coulombic efficiency is 99.6%, which is higher than that of cells containing electrolytes of disiloxane-functionalised phosphonium,^40^ allyl-functionalised piperidinium^41^ and imidazolium, pyrrolidinium, and piperidinium with film-forming additives.^42^,^43^ The results above mean an outstanding cycle stability for Li/MCMB cells with LiODFB–Pyr_1,2O1_TFSI/DMS because DMS is beneficial for SEI forming on the surface of the MCMB.

The Nyquist plots of AC impedance spectra of cells containing different electrolytes are shown in Fig. S6.† In general, the total cell resistance mainly consists of the bulk resistance (*R*_b_), the resistance to Li^+^ migration through the SEI layer (*R*_sei_), and the charge transfer resistance (*R*_ct_).^38^ It is revealed that both the *R*_b_ and *R*_ct_ values are much smaller for electrolytes with sulphites than for those of LiODFB–Pyr_1,2O1_TFSI. The *R*_ct_ value of cells containing LiODFB–Pyr_1,2O1_TFSI/ES is higher than that of cells containing LiODFB–Pyr_1,2O1_TFSI/DMS, which might be attributed to the excessively thick SEI layer formed in ES and the consequent difficulty for Li^+^ to transfer through the electrode–electrolyte interface.

A combination of density functional theory (DFT) modelling, electrochemical testing and scanning electron microscopy (SEM) analysis reveals the film-forming mechanisms of the mixed electrolytes. LiODFB can facilitate the formation of a SEI by electrochemical redox reactions.^44^ The reduction of the F_2_B[ox]^–^ anions may contribute to the formation of the inorganic inner layer of the SEI:^45^

2 F_2_B[ox]^–^ + e^–^˙ → [ox]B˙ + 2F^–^

**Fig. 4:**
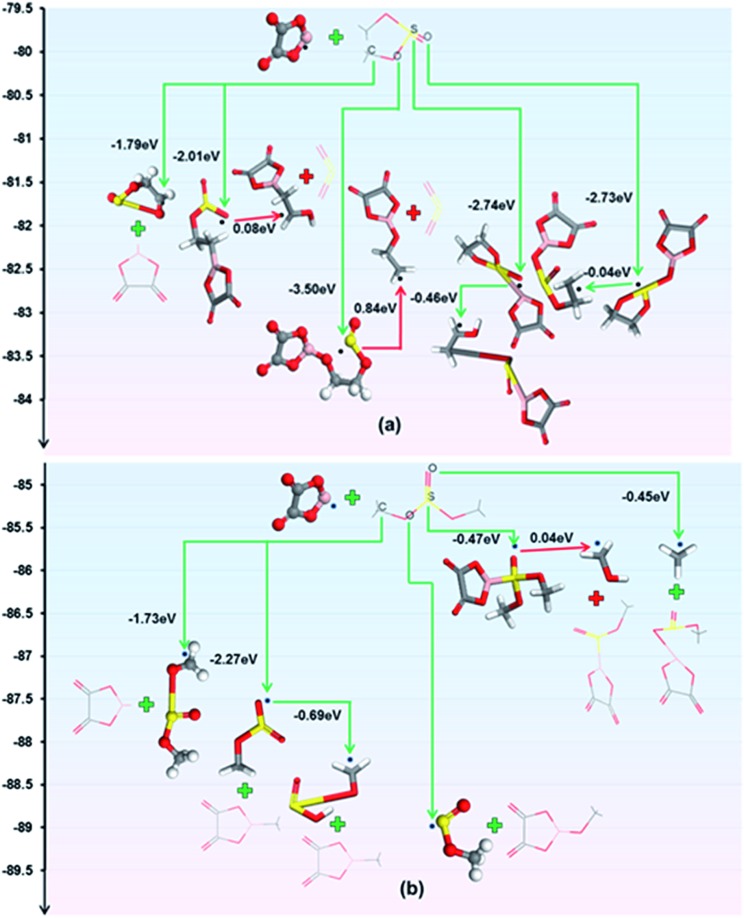
Reactions of [ox]B˙ radicals with (a) ES and (b) DMS.

The [ox]B˙ radical can react with sulphite molecules, and the reactions with the energy change obtained by DFT calculations are shown in Fig. 4. According to the principle of lowest energy, the prime reactions for ES and DMS are

3 [ox]B˙ + ES → [ox]BOCH_2_CH_2_OS˙O

4 [ox]B˙ + DMS → [ox]BOCH_3_ + CH_3_OS˙O

with an overall energy change of 3.50 eV and 3.75 eV, respectively. In addition, the SO_2_ gas which is noxious and causes the swelling of cells is negligible because the reactions are endergonic to produce the SO_2_ gas.

**Fig. 5:**
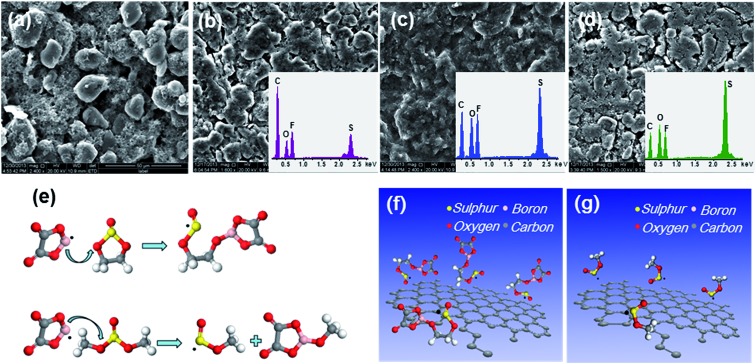
(a) SEM image of the MCMB anode before cycling. SEM and EDS of the SEI layer on the MCMB anode in (b) LiODFB–Pyr_1,2O1_TFSI, (c) LiODFB–Pyr_1,2O1_TFSI/ES, and (d) LiODFB–Pyr_1,2O1_TFSI/DMS electrolytes after 10 cycles. Schematic illustration of the synthesis route to SEI: (f) ES; (g) DMS, with the prime reactions (e).

To validate the calculation results, the SEM, energy dispersive spectrometry (EDS) and schematic illustration of the synthesis route to the SEI layer with prime reactions in the electrolytes are shown in Fig. 5. The spherically shaped particles of the original MCMB electrode are obscure, as a film covers their surface after cycling. The films formed in the electrolyte containing sulphites are more compact than those formed in LiODFB–Pyr_1,2O1_TFSI. Moreover, the film grown in the electrolyte containing ES is thicker and has a lower S content than in DMS (Table S1†), which is consistent with the calculation results.

**Fig. 6:**
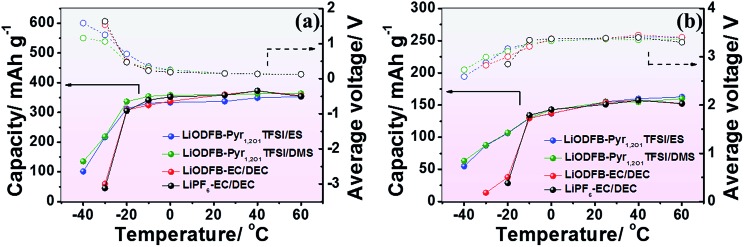
Performance of Li/MCMB (a) and Li/LiFePO_4_ cells (b) containing LiODFB–Pyr_1,2O1_TFSI/ES, LiODFB–Pyr_1,2O1_TFSI/DMS, LiODFB–EC/DEC or LiPF_6_–EC/DEC electrolytes at various temperatures with a current density of 0.1C.

The olivine LiFePO_4_ has been developed with a low toxicity, low cost and high safety^46^ as an electrode material for lithium-ion batteries. However, Li/LiFePO_4_ containing the traditional electrolyte, cannot operate at low temperatures below –20 °C according to observations reported by Wang Y.^46^Fig. 6 and S7† show the electrochemical performance of the Li/MCMB cells and Li/LiFePO_4_ cells in LiODFB–Pyr_1,2O1_TFSI/ES, LiODFB–Pyr_1,2O1_TFSI/DMS, the traditional electrolyte (1 M LiPF_6_–EC/DEC (1 : 1 by volume)) and the 1 M LiODFB–EC/DEC (1 : 1 by volume) at temperatures ranging from –40 °C to 60 °C. The cells with the traditional electrolyte exhibit poor low temperature performance. LiODFB provides some improvement in the electrochemical performance of the lithium-ion cells at low temperatures,^47^ but not great breakthroughs when the carbonate species is solidified below –30 °C. The electrolytes based on ionic liquids operate well at temperatures higher than 25 °C because of their thermal stability,^48^,^49^ but suffer from the same problem as traditional electrolytes at low temperatures. Improvement by adding sulphites in ether-functional ionic liquid electrolytes enables operation at lower temperatures. The capacities of Li/MCMB and Li/LiFePO_4_ cells containing LiODFB–Pyr_1,2O1_TFSI/DMS are 354.2 mA h g^–1^ and 130.3 mA h g^–1^ at –10 °C with average voltages of 0.306 V and 3.283 V, respectively. Additionally, these cells are able to operate with acceptable electrochemical performance at temperatures as low as –40 °C.

**Fig. 7:**
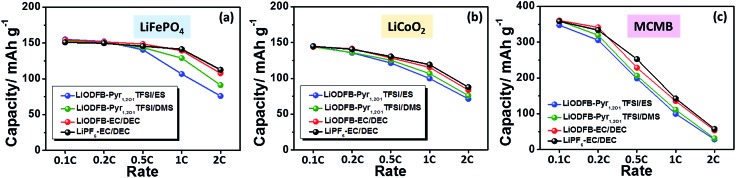
Rate capability of the Li/LiFePO_4_ (a), Li/LiCoO_2_ (b) and Li/MCMB (c) cells with LiODFB–Pyr_1,2O1_TFSI/ES, LiODFB–Pyr_1,2O1_TFSI/DMS, LiODFB–EC/DEC or LiPF_6_–EC/DEC electrolytes.

LiCoO_2_ has been widely used in lithium-ion batteries as a cathode material. To ensure the effect of the developed electrolyte on the electrochemical performance of lithium-ion cells, the rate capability of the LiODFB–Pyr_1,2O1_TFSI/DMS electrolyte in Li/LiFePO_4_, Li/LiCoO_2_ and Li/MCMB cells are shown in Fig. 7. The discharge capacities for the Li/LiFePO_4_ and Li/LiCoO_2_ cells containing LiODFB–Pyr_1,2O1_TFSI/DMS at 1C (150 mA g^–1^) are around 129 and 106.7 mA h g^–1^, respectively, and the charge capacity for the Li/MCMB cells at 0.5C (150 mA g^–1^) is about 206.5 mA h g^–1^. A good rate performance depends on both satisfactory ion transport and acceptable interfacial reaction resistance in the cells.

### Ring-chain synergy

**Fig. 8:**
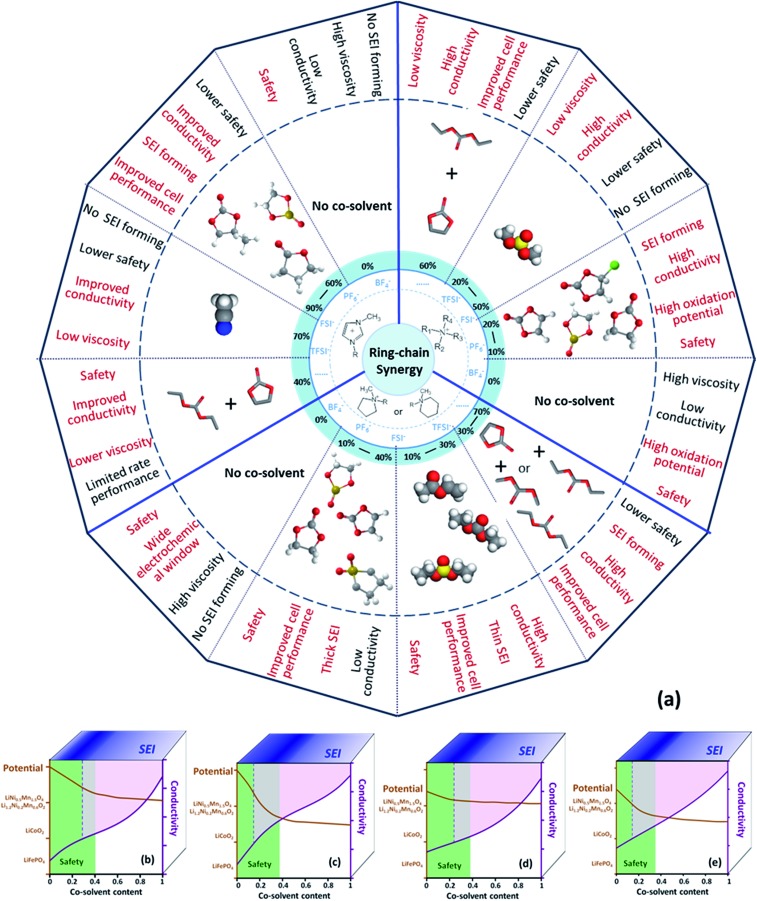
(a) Illustration of representative electrolyte systems based on ionic liquids for lithium batteries with the corresponding characteristics. Semi-quantitative threshold of the properties of the electrolyte systems: (b) ring-like ionic liquid and ring-like cosolvent, (c) ring-like ionic liquid and chain-like cosolvent, (d) chain-like ionic liquid and ring-like cosolvent and (e) chain-like ionic liquid and chain-like cosolvent.^11^,^13^–^17^,^19^,^24^,^34^,^46^–^78^

To clarify if the ring-chain synergy in the carbonate electrolytes used in lithium-ion batteries is also applicable for the ionic liquid electrolytes, a summary of representative electrolyte systems of ionic liquids with organic cosolvents for lithium-ion batteries and the corresponding characteristics and properties are given in Fig. 8.^11^,^13^–^17^,^19^,^24^,^34^,^46^–^78^ The relationship among the structures of electrolyte components and their properties is shown to provide a good starting point for strategically designing new electrolyte systems. Furthermore, the comparisons of the improvements offered by the ring-like ES and chain-like DMS in the performance of the electrolytes based on Pyr_1,2O1_TFSI offer some support for this relationship.

Generally, a safe electrolyte should satisfy several requirements such as non-flammability, good ion transport and compatibility with both the cathode and anode in lithium-ion batteries. The electrolytes based on imidazolium are electrochemically unstable at high potential.^79^ Therefore we have focused on ammonium-based electrolytes.

#### Safety

Safety is related to the flammability of the electrolyte and can be improved using combined ionic liquid/cosolvent electrolyte systems. For the electrolyte systems investigated in the present work, the non-flammability is retained when the amount of cosolvent added is kept below approximately 40 wt%. The electrolytes containing chain-like components require higher concentrations of ionic liquid, as the chain-like cosolvent is more thermally unstable.^80^

#### Ion transport

In this study, the ionic conductivity of the electrolyte with DMS is higher than that with ES because the chain-like DMS possesses a lower viscosity, which is beneficial for ionic mobility. A satisfactory electrolyte for a battery cell should have a Li^+^ conductivity greater than 10^–1^ mS cm^–1^.^81^ Moreover, in order for the cell to show a good rate performance, the electrolyte should show an ionic conductivity above 1 mS cm^–1^ at room temperature. To reach this target, the amount of ring-like and chain-like cosolvents introduced into the ring-like ionic liquid pyrrolidinium or piperidinium should be approximately 25–35 wt% or 15–25 wt%, respectively. The most conventional tetra-alkylammonium cations are seldom selected to form electrolytes for lithium batteries, mainly because of their high melting temperatures.^82^ However, an increasing molecular weight will lead to stronger van der Waals forces between molecular ionic liquids, resulting in increased viscosity.^83^,^84^ As a result, the amount of cosolvents in chain-like ammonium required to reach a conductivity of 1 mS cm^–1^ is lower than that in ring-like pyrrolidinium or piperidinium.

#### Compatibility with cathodes

The electrochemical window is an important index related to the compatibility of the electrolyte with the electrodes in a battery cell. A high-voltage cathode material requires an electrolyte with a high oxidation potential, which restricts the composition of the electrolyte systems. As shown in Fig. 2(g), the energy of the LUMO and HOMO of ES is lower than that of DMS, indicating higher oxidation and reduction potentials. The results illustrate that ES is a more suitable cosolvent for high-potential cathodes and is more prone to decompose on the carbonaceous anode. Most of the electrolytes containing both components with ring-like structures can meet the demands of high-potential cathodes; this may also be achieved when the chain-like cosolvents account for less than approximately 25%; for chain-like ionic liquid systems, the contents of ring-like cosolvents should be less than approximately 40%; however, this demand is difficult to meet using mixed electrolytes in which both components have chain-like structures.

#### Compatibility with anodes

Carbon is widely used as an anode which requires cosolvents with a suitable reduction potential to form the SEI layer. In this study, both higher capacities and better cycle stability are obtained in Li/MCMB cells with an electrolyte containing DMS rather than ES. This could be due to one of two reasons: (1) the reduction peak corresponding to the formation of an SEI with the electrolyte containing DMS is weaker, indicating a thinner SEI layer; (2) the electrolyte with DMS has a low viscosity and high separator wettability. An excessively thick SEI layer would result in a high resistance, making it difficult for Li^+^ to transfer through the electrode–electrolyte interface. Moreover, the calculated [ox]B˙ radical releases more energy when reacting with DMS than with ES, so DMS is more prone to form an SEI. Meanwhile, a larger mass of product is introduced by the reduction of ES, making the SEI thicker. These results confirm that in electrolytes containing a ring-like ionic liquid, the chain-like DMS is more beneficial as the cosolvent than the ring-like ES. An optimal proportion of cosolvents should be chosen such that they form a SEI layer that is neither too thick (leading to high resistance in the battery) nor too thin (providing no protection for the electrodes).

General performances of the electrolytes based on ionic liquids with cosolvents are divided into four factors: safety, conductivity, oxidation potential and SEI forming ability. They depend on the nature of the ionic liquids and the cosolvents. We tried to suggest how the electrochemical performances of the electrolyte systems varied with the contents of the cosolvents through analyzing research data from this work and previous studies and the results are shown in Fig. 8(b)–(e). For example, Fig. 8(b) exhibits the general performance of a mixed electrolyte based upon a ring-like ionic liquid with ring-like cosolvents. The applicable content of the cosolvent should be less than 40 wt% for non-flammability of the bulk electrolyte (green zone), because the electrolyte with 40 wt% ring-like cosolvent and 60 wt% ring-like ionic liquid was just beginning to become non-flammable.^19^ Cosolvents were added to an ionic liquid to increase the room temperature ionic conductivity of the bulk electrolyte to over 1 mS cm^–1^. The amount of cosolvents added depends on the nature of the specific components. For systems exhibited in the present and previous studies, it should be more than 25–35 wt% (purple zone).^19^,^49^,^53^,^54^ The oxidation potentials of the investigated electrolyte systems containing a ring-like ionic liquid and a ring-like cosolvent are higher than 5 V (brown line), which can meet the requirements of the cathodes widely used in lithium-ion batteries such as LiFePO_4_, LiCoO_2_, LiNi_1/3_Mn_1/3_Co_1/3_O_2_, and Li_1.2_Ni_0.2_Mn_0.6_O_2_. Besides, the contents of the cosolvents should be chosen to form a suitable SEI layer to protect the carbonaceous anodes, which is about 20 wt% (blue zone) in accordance with the previous studies. The performances of the electrolyte systems containing “ring-like ionic liquid and chain-like cosolvent”, “chain-like ionic liquid and ring-like cosolvent” and “chain-like ionic liquid and chain-like cosolvent” are analogously summed up to search for the optimal proportion of the electrolyte components on the basis of this work and the reported studies.

## Conclusions

Study of the electrolytes based on the ring-like Pyr_1,2O1_TFSI with ES and DMS reveals that the sulphites improve the electrolyte conductivity, compatibility with MCMB electrodes and cell performance at low temperatures. DMS with a lower viscosity and melting point can better enhance the cell performance than ES in the electrolyte with the main component of Pyr_1,2O1_TFSI. The conductivity of the LiODFB–Pyr_1,2O1_TFSI/DMS electrolyte was 8.163 × 10^–3^ S cm^–1^. The Li/MCMB and Li/LiFePO_4_ cells containing this electrolyte had good cycle performance and a normal charge curve in the temperature range of –40 °C to 60 °C.

Furthermore, this research identifies the relationship between ionic liquid/cosolvent electrolyte performances and the component structures. According to this work and previous studies, ionic liquid electrolyte systems consisting of both a ring-like component and a chain-like component in a specific ratio can offer good overall battery performance – measured in terms of safety, ionic conductivity and compatibility with cathode and anode materials. The ring-chain synergy that takes advantage of the availability of the two structures might offer a useful starting point to design mixed electrolytes in lithium-ion batteries. In addition, electrolytes with both ring-like components and chain-like components might be developed for applications under particular conditions due to their outstanding electrochemical window and ionic conductivity, respectively. The insight into the ring-chain synergy will allow further anticipation of the ionic liquid/cosolvent electrolytes. It is noteworthy that only the chemical structures of the cosolvent and the cations of ionic liquid were taken into consideration here. In order to gain a better understanding of a broader range of systems and to achieve an even better performance, further study should explore functional modifications and the effect of anions, which might further improve the properties of the electrolyte systems.

## Supplementary Material

Supplementary information
