# Supplementary material for: Ring-chain synergy in ionic liquid electrolytes for lithium batteries
Source: Chem Sci. 2015 Sep 18;6(12):7274–83. doi: 10.1039/c5sc02761f (PMC5950758; doi:10.1039/c5sc02761f)
Supplement: Supplementary file 1 [file SC-006-C5SC02761F-s001.pdf]

Electronic Supplementary Material (ESI) for Chemical Science.
This journal is © The Royal Society of Chemistry 2015

Electronic Supplementary Material (ESI)

Ring-Chain Synergy in Ionic Liquid Electrolytes for Lithium Batteries

Feng Wu[a,b], Qizhen Zhu[a], Renjie Chen[a,b,*], Nan Chen[a], Yan Chen[a], Li Li[a,b]

[a] *School of Chemical Engineering and Environment, Beijing Institute of Technology, Beijing Key Laboratory of Environmental Science and Engineering, Beijing 100081, China*

[b] *National Development Center of High Technology Green Materials, Beijing 100081, China*

*Corresponding author at: School of Chemical Engineering and Environment, Beijing Institute of Technology, Beijing Key Laboratory of Environmental Science and Engineering, Beijing 100081, China. Tel.: +86 10 68912508; fax: +86 10 68451429.

E-mail addresses: chenrj@bit.edu.cn (R. Chen).

Experimental Methods

Materials.

ES (99%, J&K Scientific Ltd), DMS (99%, J&K Scientific Ltd), and $Pyr_{1,2O1}$TFSI (99%, Shanghai Chengjie Chemical Co. LTD) were used after dehydration for 12 h. LiODFB (0.4 M) was dissolved in $Pyr_{1,2O1}$TFSI with and without sulphites in an argon-filled glove box to prepare the three systems of composite electrolytes: 0.4 M LiODFB in $Pyr_{1,2O1}$TFSI, 0.4 M LiODFB in $Pyr_{1,2O1}$TFSI:ES (7:3 by weight), and 0.4 M LiODFB in $Pyr_{1,2O1}$TFSI:DMS (7:3 by weight). Water content of the electrolyte systems were less than 20 ppm.

Preparation of electrodes and cell manufacture.

Anodes used in electrochemical half-cells of Li/MCMB (or Li/$LiFePO_4$, Li/$LiCoO_2$) consisted of 80 wt% active material, 10 wt% acetylene black, and 10 wt% polyvinylidene fluoride. The mixed slurry was coated onto the current collector, dried at 70 °C under vacuum and cut into pellets as the size of diameter 8mm and the areal loading of about 4.5mg cm^{-2}. Electrochemical half-cells were assembled using a Celgard®2300 separator and with the prepared electrolytes in the argon-filled glove box.

Instruments and characterizations.

The IR spectra of the electrolyte samples were recorded on a NICOLET6700 spectrometer at 4000 cm^{-1} – 400 cm^{-1} under an Ar atmosphere. The ionic conductivity and the electrochemical impedance spectra (EIS) were measured using a CHI660D electrochemical workstation over the test frequency range of 10^5~10 Hz and 10^5~10^{-2} Hz, respectively. The electrochemical windows for various electrolytes sealed in a glass cell with a platinum wire as the working electrode and Li foil as the reference and counter electrodes were tested by cyclic voltammetry (CV) on a electrochemical workstation in the voltage range of –0.5 V to 6.0 V at a scan rate of 0.1 $mV \cdot s^{-1}$. Constant current charge-discharge experiments were

carried out on a Land cell tester at a current density of 0.1C (30 mA g^{-1} for the anodes and 15 mA g^{-1} for the cathodes), and the operating temperature range was set in –40 °C – 60 °C. For Li/MCMB cells, the operation voltages were set at 2V-0.005V. For Li/$LiCoO_2$ and Li/$LiFePO_4$ cells, the operation voltages were set at 4.2V-2.7V and 4.2V-2.5V respectively. The morphology and chemical composition of the surface of the cycled MCMB anode were characterised by SEM and EDS. The calculations were performed with the DMol3 module of the Materials Studio 5.5 program. The sulphite molecules and radical structures were optimised and calculated employing nonlocal DFT with the BLYP functional based on a DNP group. A DSC measurement was performed to investigate thermal behaviour using a MDSC 2910 DSC. The flammability of the electrolytes was tested by immersing a glass fibre ball into the electrolyte and then exposing it to a butane blowtorch flame. The electrolyte was judged to be non-flammable if the electrolyte failed to ignite within a testing time of 60 s. If the electrolyte was burning, the blowtorch was removed and the self-extinguishing time was recorded. Each electrolyte was tested five times.

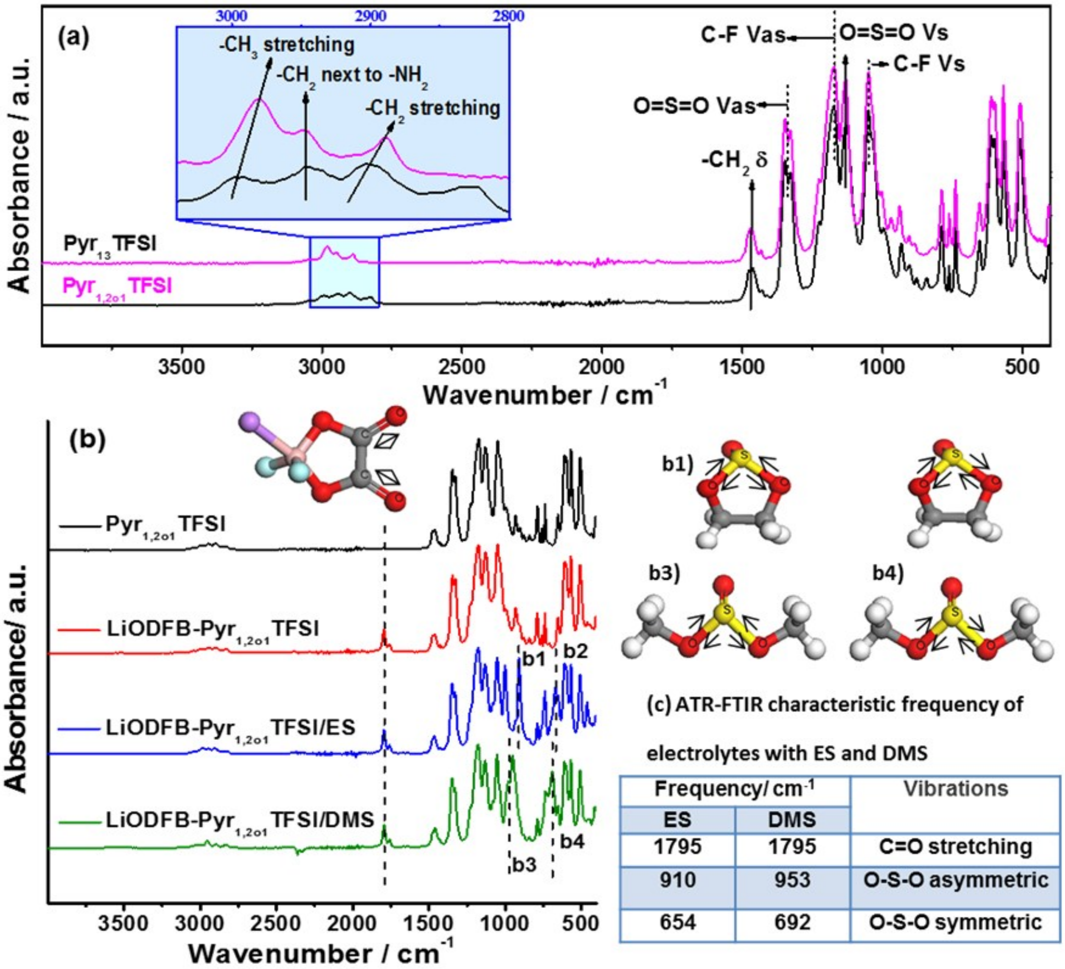

Frequency/ cm-1		Vibrations
ES	DMS	
1795	1795	C=O stretching
910	953	O-S-O asymmetric
654	692	O-S-O symmetric

Figure S1. The ATR-FTIR spectra of $Pyr_{13}TFSI$ and $Pyr_{1,2O1}TFSI$ with and without LiODFB and sulphites.

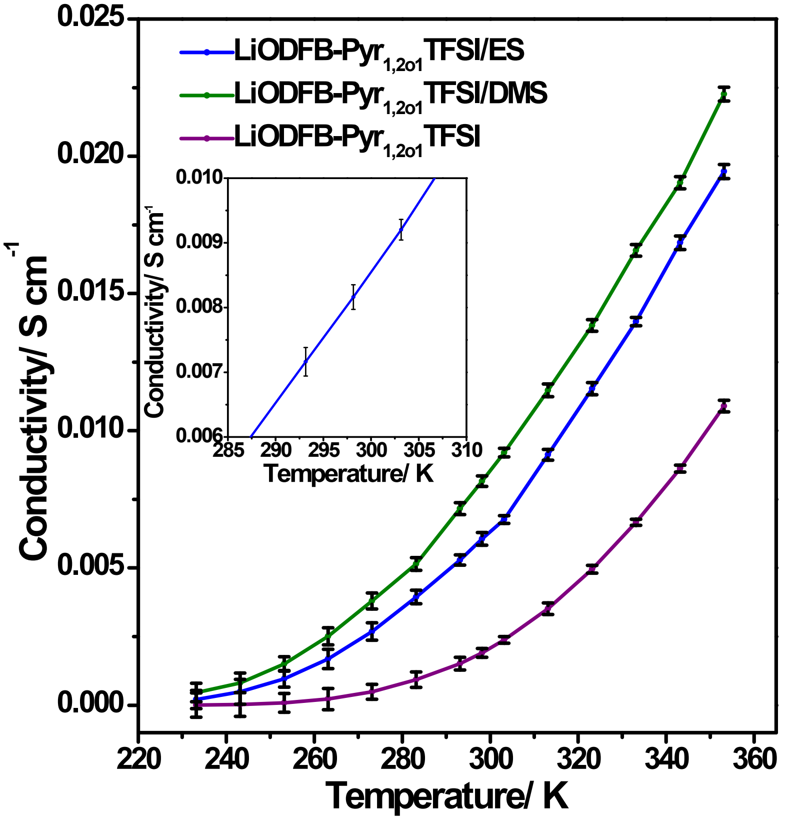

Figure S2. Ionic conductivity with error bar of the LiODFB-$Pyr_{1,2O1}$TFSI electrolytes with and without sulfites at various temperatures; More detail about the ionic conductivity with error bar of LiODFB-$Pyr_{1,2O1}$TFSI/DMS in 285-310K is given in the insert.

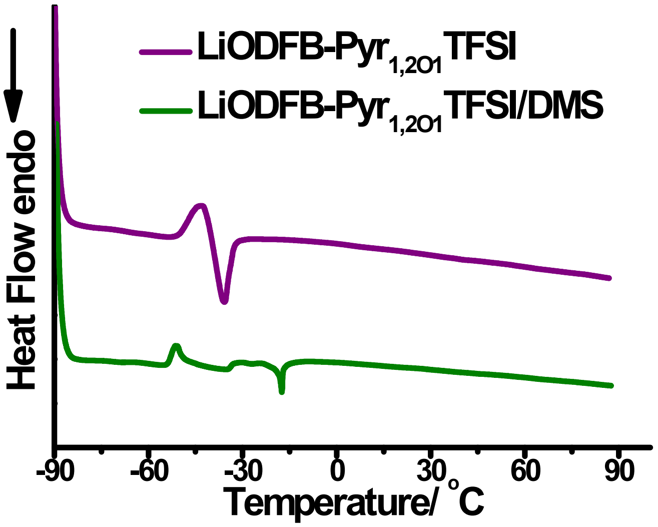

Figure S3. DSC curves of LiODFB-$Pyr_{1,2O1}$TFSI and LiODFB-$Pyr_{1,2O1}$TFSI/DMS.

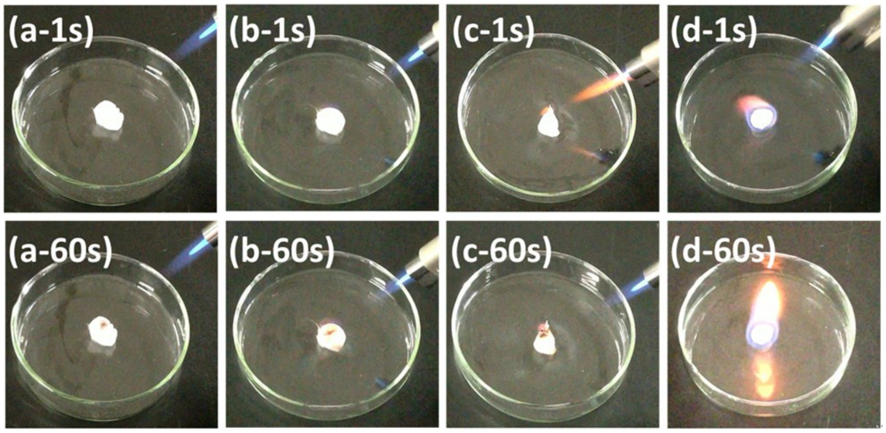

Figure S4. Burning tests of (a) LiODFB-$Pyr_{1,2O1}$TFSI, (d) LiODFB-$Pyr_{1,2O1}$TFSI/ES, (c) LiODFB-$Pyr_{1,2O1}$TFSI/DMS and (d) $LiPF_6$-EC/DEC.

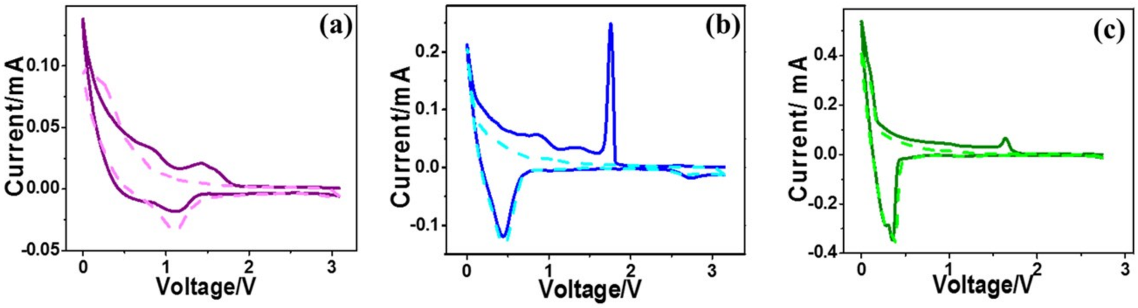

Figure S5. CVs of Li/MCMB cells containing (a) LiODFB-$Pyr_{1,2O1}$TFSI, (b) LiODFB-$Pyr_{1,2O1}$TFSI/ES and (c) LiODFB-$Pyr_{1,2O1}$TFSI/DMS.

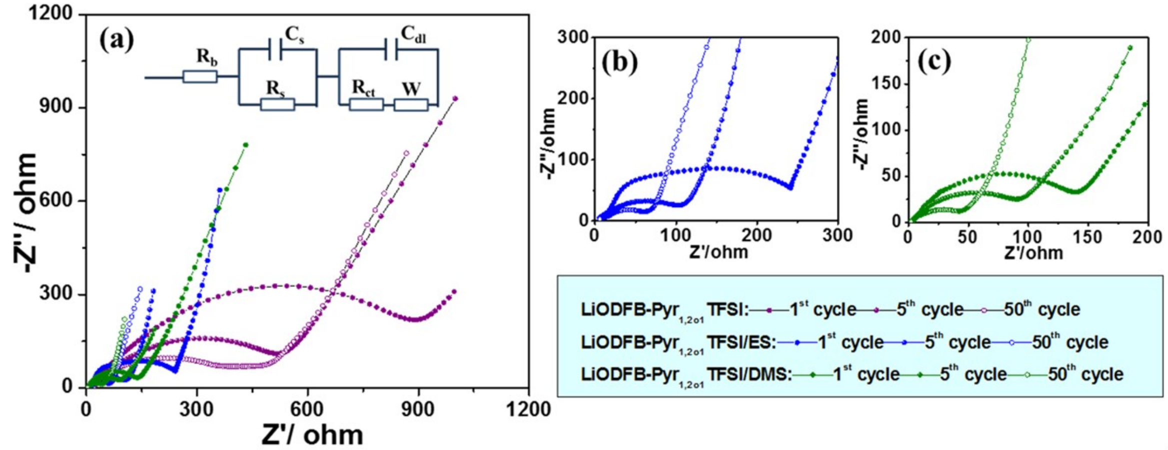

Figure S6. (a) Nyquist plots of the AC impedance spectra of the three types of cells and equivalent circuit; more details about cells with LiODFB-$Pyr_{1,2O1}$TFSI/ES and LiODFB-$Pyr_{1,2O1}$TFSI/DMS are given in (b) and (c).

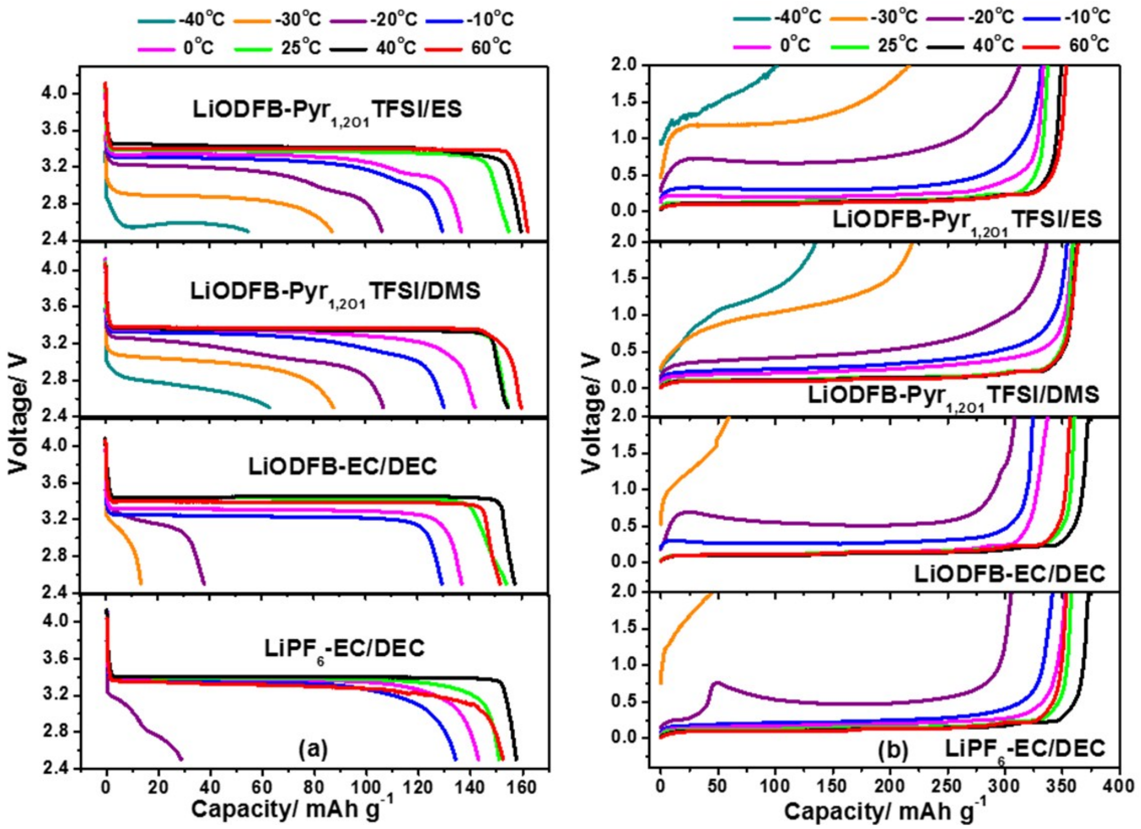

Figure S7. Performance of Li/MCMB (a) and Li/$LiFePO_4$ cells (b) containing LiODFB-$Pyr_{1,2O1}$TFSI/ES, LiODFB-$Pyr_{1,2O1}$TFSI/DMS, LiODFB- EC/DEC or $LiPF_6$-EC/DEC electrolytes at various temperatures with a current density of 0.1C.

Table S1. Element mass fraction of the electrolyte.

Element	LiODFB-$Pyr_{1,2o1}$TFSI	LiODFB-$Pyr_{1,2o1}$TFSI/ES	LiODFB-$Pyr_{1,2o1}$TFSI/DMS
Carbon	53.72wt%	36.21wt%	34.44wt%
Oxygen	15.3wt%	20.79wt%	23.62wt%
Fluorine	23.54wt%	28.56wt%	24.05wt%
Sulfur	3.94wt%	8.4wt%	12.28wt%
